# Pediatric COVID-19: Low Incidence, but Possible Fatality—A Case Report and a Review of the Literature

**DOI:** 10.3390/children8121128

**Published:** 2021-12-03

**Authors:** Cristina Oana Mărginean, Lorena Elena Meliț, Iunius Simu, Claudiu Puiac, Janos Szederjesi, Maria Oana Săsăran

**Affiliations:** 1 Department of Pediatrics I, George Emil Palade University of Medicine, Pharmacy, Science, and Technology of Târgu Mureș, Gheorghe Marinescu Street No 38, 540136 Târgu Mureș, Romania; marginean.oana@gmail.com; 2 Department of Radiology, George Emil Palade University of Medicine, Pharmacy, Science, and Technology of Târgu Mureș, Gheorghe Marinescu Street No 38, 540136 Târgu Mureș, Romania; iuniuspaul.simu@gmail.com; 3 Department of Emergency Medicine, George Emil Palade University of Medicine, Pharmacy, Science, and Technology of Târgu Mureș, Gheorghe Marinescu Street No 38, 540136 Târgu Mureș, Romania; claudiupuiac@gmail.com; 4 Department of Anestesiology, George Emil Palade University of Medicine, Pharmacy, Science, and Technology of Târgu Mureș, Gheorghe Marinescu Street No 38, 540136 Târgu Mureș, Romania; yangzi37@gmail.com; 5 Department of Pediatrics III, George Emil Palade University of Medicine, Pharmacy, Science, and Technology of Târgu Mureș, Gheorghe Marinescu Street No 38, 540136 Târgu Mureș, Romania; oanam93@yahoo.com

**Keywords:** SARS-CoV-2, COVID-19, coagulopathy, pneumonia, children

## Abstract

Background: Pediatric COVID-19 is a current health burden mostly due to the lack of knowledge in terms of symptoms, clinical course and management. COVID-19-associated coagulopathy is one of the most recently described complications among adults, along with acquired thrombophilia resulting in an increased risk for venous, arterial and microvascular thrombosis. Case presentation: We report the case of a 4-year-old male child, admitted to our clinic for generalized seizures being intubated and mechanically ventilated before admission, with a personal history of ureterovesical junction obstruction, mild hydronephrosis, and an episode of generalized seizures. The laboratory tests revealed anemia, an increased number of monocytes, and a mildly increased C-reactive protein. A real-time polymerase chain reaction (RT-PCR) of the oropharyngeal swab was performed and it tested positive for SARS-CoV-2 in the child and both of his parents. The thoracic CT showed consolidation in the lower lobe of the left lung associated with an opacity in the right apex, suggesting possible atelectasis. We initiated antibiotic, antiviral, corticosteroids, as well as anticoagulants and antipyretics, continuing the chronic anticonvulsant therapy. The patient’s condition deteriorated progressively, and, after 72 h of hospitalization, he developed desaturation and bradycardia. The laboratory parameters on the third day showed leucopenia, neutropenia, increased creatine kinase, a high ferritin level, hypoalbuminemia, a prolonged prothrombin time and an increased international normalized ration. The patient died on the fourth day of admission. Conclusion: In spite of its low incidence and frequent benign clinical course, COVID-19 complications such as coagulopathy might represent a leading cause of death, even in pediatric patients.

## 1. Introduction

Coronavirus disease 2019 (COVID-19) is the most recent infectious disease, emerging in December 2019 in Wuhan, China, and caused by severe acute respiratory syndrome coronavirus 2 (SARS-CoV-2). It has quickly become a global burden for both clinicians and healthcare systems [1]. Unfortunately, we are not nearly close to the end of this pandemic and healthcare providers continue to be overwhelmed in most countries worldwide. Each day of the pandemic reveals additional challenges in daily practice and there are still multiple knowledgegaps in terms of pathogenesis, clinical picture and standardized protocol of treatment. It was noticed that COVID-19 especially affects adults, but the incidence of this condition in children increased over time, reaching up to 8.8% of the total cases in USA, according to CDC reports updated on the 10th of October [2]. The CDC reported that the cases diagnosed between 0–4 years accounted for 1.9%, while those between 5–17 years for 6.9%. Nevertheless, the deaths between 0–4 years and 5–17 years were encountered in less than 0.1% of the patients. In Europe, 6,918,265 million people were confirmed to have COVID-19 as of 10th of October 2020, resulting in 246,709 deaths. Out of these COVID-19-related deaths in Europe, 5358 were in Romania, accounting for an alarming mortality rate of 2.17% [3]. Fortunately, children represented only 6% of all confirmed cases in Romania, with 3526 cases in the 0–9 years age group and 6337 in the 10–19 years age group [4]. Surprisingly, only one death caused by COVID-19 was reported in Romania up to this date in the age group of 10–19 years. Therefore, Romania follows the pattern reported worldwide in terms of COVID-19 severity in pediatric patients. There are multiple hypotheses explaining the lower incidence and milder forms in children, such as: (1) the immaturity of the immune system; (2) the improper distribution, maturation and functioning of the angiotensin-converting enzyme 2; or (3) the presence of other viral infection that might limit the replication of SARS-CoV-2 [5,6,7].

The clinical spectrum of children with COVID-19 varies from asymptomatic, the most common situation, to life-threatening forms in rare cases. The scarcity of COVID-19 data in pediatric patients results from an increased frequency of asymptomatic cases and subsequent low rate of testing in this age group. Thus, a large pediatric review underlined that 13% of patients with a virologically confirmed SARS-CoV-2 infection were asymptomatic [8]. In those who experienced symptoms, the most frequently encountered were fever, cough, sore throat, sneezing, wheezing, myalgia, and fatigue [8]. Gastrointestinal symptoms might also appear in patients with this novel infection, with a loss of appetite being more common among adults, while diarrhea might be encountered in both adults and children [9]. The prevalence of life-threatening forms of COVID-19 in children seems to be inversely related to age, according to the findings of Dong et al., who underlined that half of the children with critical forms of COVID-19 were between 0 and 1 years old [8]. COVID-19-associated coagulopathy is one of the most recently described complications of this infection among adults, along with acquired thrombophilia, resulting in an increased risk for venous, arterial and microvascular thrombosis [10]. 

Therefore, we report the case of the youngest child who died of COVID-19 and the second pediatric COVID-19-related death from Romania, as of October 2020. This underlines that, despite the lower incidence of COVID-19 in children compared to adults, this infection might result in severe forms and life-threatening complications, such as coagulopathy and even death. 

The written informed consent was obtained from the patient’s mother prior to publication of this case. 

## 2. Case Report

### 2.1. Presenting Concerns

We report the case of a 4-year-old male child, admitted to our clinic for generalized seizures, which persisted in spite of anticonvulsant therapy (Diazepam), with no previous acute symptoms. His personal history revealed ureterovesical junction obstruction, mild hydronephrosis, and an episode of generalized seizures approximately 2 months before the current admission for which chronic therapy with sodium valproate (Depakine) was recommended. We must mention that the brain MRI performed at that time was normal. The family history showed the presence of ageusia and anosmia in both parents. 

### 2.2. Clinical Findings

At the time of admission, the patient was intubated and mechanically ventilated and the clinical exam revealed only pallor. 

### 2.3. Diagnostic Focus and Assessment

**Figure 1:**
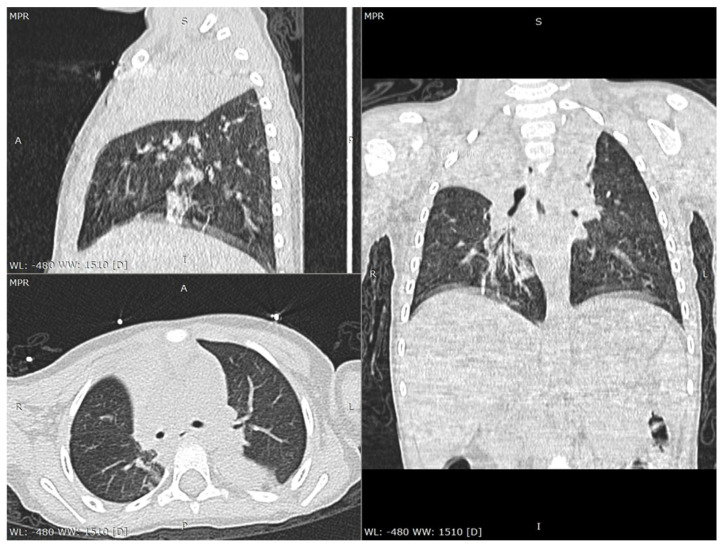
Chest CT scan lung window: multiplanar reconstructions (axial, sagittal and coronal views) show atelectatic consolidation of the right upper lobe.

**Figure 2:**
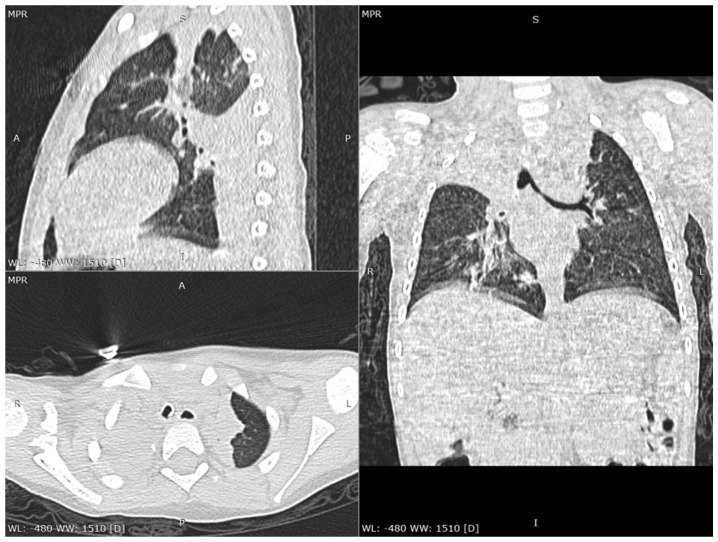
Chest CT scan lung window: multiplanar reconstructions (axial, sagittal and coronal views) also show atelectasis of several segments in the left lung (ventral segment from upper lobe, apical and posterior segments from lower lobe).

The laboratory tests performed on the day of admission revealed anemia (Hemoglobin—Hb 9.98 g/dL, Hematocrit—Htc 28.54%), a severely increased number of monocytes (9624/µL), and a mildly increased C-reactive protein (CRP 7 mg/L). Taking into account the family history, a real-time polymerase chain reaction (RT-PCR) of the oropharyngeal swab was performed and it tested positive for SARS-CoV-2. Moreover, both parents were confirmed with this infection. Both urine and blood cultures were negative. The serology for viral hepatitis B and C, as well as antinuclear and anti-double-strained DNA antibodies were negative. We performed a thoracic computed tomography (CT), which showed consolidation in the lower lobe of the left lung associated with an opacity in the right apex, suggesting possible atelectasis (Figure 1 and Figure 2). The cranial CT revealed no pathological findings. The patient was admitted to the intensive care unit with a diagnosis of COVID-19 in a severe form. 

### 2.4. Therapeutic Focus and Assessment

We initiated antibiotic treatment (ceftriaxone 800 mg twice a day and amikacin 100 mg twice a day), antiviral therapy (lopinavir/ritonavir 2.5 mL twice a day), corticosteroids (Dexamethasone 4 mg twice a day), anticoagulants (enoxaparin 0.2 mL in a single daily dose), and antipyretics (Paracetamol), and we continued the chronic anticonvulsant therapy with sodium valproate. The second RT-PCR performed on the third day of admission was also positive for SARS-CoV-2 infection. Unfortunately, the patient’s condition deteriorated progressively, and, after approximately 72 h of hospitalization, he developed desaturation and bradycardia. We repeated the laboratory parameters before the bradycardia event and we found leucopenia (leukocytes 3500/µL), neutropenia (neutrophils 1111/µL), mildly increased creatin kinase (280 U/L), a high ferritin level (121 ng/mL, normal ranges 7–84 ng/mL), hypoalbuminemia (3.29 g/dL), a prolonged time of prothrombin (22.3 s) and an increased international normalized ration (INR 1.74). 

### 2.5. Follow-Up and Outcome

Despite all efforts to resuscitate the patient, he died on the fourth day of admission. 

## 3. Discussions

The incidence of COVID-19 in pediatric patients is reported to be lower in comparison to adults. A large review from China revealed that, out of 44,672 patients who tested positive for SARS-CoV-2, only 2% were aged between 0–19 years [11]. The reports from other countries worldwide showed an incidence between 1 and 5% and an even lower hospitalization rate in the case of children diagnosed with this condition [12,13]. Moreover, severe forms of COVID-19 express a low prevalence in this age group according to a large review performed by Dong et al.: 10.6% under the age of 1 year, 7.3% for children 1–5 years, 4.2% for 6–10 years, 4.1% for 11–15 years, and only 3% for teenagers 16–17 years [8]. These findings suggest a particular course of COVID-19 in children mostly due to their age-related peculiarities [5,6,7].

In spite of the fact that the lack of symptoms is relatively common in children, a wide spectrum of respiratory and digestive symptoms might be encountered in children with SARS-CoV-2, among which cough, pharyngeal erythema, fever, dyspnea, cyanosis, loss of appetite, diarrhea, gastrointestinal bleeding, abdominal pain, nausea or vomiting [8,14,15]. Nevertheless, according to the mother, our patient was completely asymptomatic before the seizures, in spite of the thoracic CT findings that definitely required a certain amount of time to appear. Laboratory parameters are usually unspecific for the diagnosis of SARS-CoV-2 infection, according to a review performed on 66 children, which revealed that 69.2% were found with a normal leukocyte count, 6% presented neutropenia, 4.6% neutrophilia, and 3% lymphopenia, while an increased CRP level was encountered in 13.6% of the cases [16]. The initial laboratory parameters in our cases showed only anemia, monocytosis, and a mildly increased C-reactive protein. Nevertheless, once the patient’s clinical course worsened, he developed leukopenia and neutropenia, along with hypoalbuminemia, also expressing mildly increased levels of ferritin and creatin kinase. Radiological findings might contribute to the diagnosis, revealing multilobar involvement, a peripheral distribution of lung lesions, consolidations with a surrounding halo and glass opacities, which are commonly seen on chest CT, and present in one-third of COVID-19 pediatric patients [14,17]. The chest CT performed in our case revealed consolidation within the inferior left lobe and an opacity in the right apex suggesting a multilobar involvement and peripheral distribution of the lesions. 

COVID-19 coagulopathy is likely the most severe complication that might occur as a result of this novel condition, only being reported in adult patients until now. Thus, venous and arterial thrombosis, including deep venous thrombosis, pulmonary embolism, ischemic stroke, systemic arterial thrombosis and myocardial infarction were reported as possible events in COVID-19 patients [10]. A study performed on 184 patients with COVID-19 pneumonia reported an incidence of thrombotic events of 31%, among which 27% were pulmonary embolisms, all patients being admitted in the intensive care unit and administered standard thromboprophylaxis with low-molecular-weight heparin [18]. The authors proved that thrombotic events might be predicted by older ages and suggestive of screening blood tests, such as prothrombin for a time over 3 s above the upper normal limit, and activated thromboplastin time for a time over 5 s above the upper limit of normal ranges [18]. Moreover, Tang et al. underlined that abnormal coagulation parameters, such as an increased D-dimers levels, prolonged prothrombin time, increased activated thromboplastin time and fibrinogen degradation products, representing predictors of poor prognosis, and frequently encountered in COVID-19 pneumonia non-survivors [19]. Additionally, the changes in these parameters persisted after hospitalization, with fibrinogen concentrations and antithrombin activity becoming normal over time in certain patients [19]. Another study, which included 150 patients admitted to the intensive care unit with COVID-19 and acute respiratory distress syndrome (ARDS), revealed a significantly higher incidence of thromboembolic events in these patients compared to those with non-COVID-19, infection-associated ARDS [20]. Anticoagulant therapy, i.e., low-molecular-weight heparin administered for at least 7 days was proven to be associated with a lower 28-day mortality according to a study performed on 445 patients with COVID-19-induced sepsis [21]. Consumptive coagulopathy and hypercoagulability are also possible in the most severe cases of COVID-19, requiring extracorporeal membrane oxygenation [22]. Our patient most likely presented COVID-19-pneumonia-associated coagulopathy despite the early, initiated, preemptive anticoagulant therapy, considering the prolonged prothrombin time and increased INR. Unfortunately, due to the rapid deterioration of our patient, we were not able to perform additional tests, such as D-dimers, fibrinogen or activated thromboplastin time. The autopsies of four patients confirmed with SARS-CoV-2 infection, who were associated with increased fibrinogen, ferritin, D-dimers levels and a prolonged prothrombin time revealed diffusely edematous lung parenchyma, peripheral hemorrhage within the lung parenchyma, and the presence of small and firm thrombi within the parenchyma on sections [23]. In addition to the prolonged prothrombin time and increased INR, our patient also encountered a higher level of ferritin, emphasizing once more the presence of COVID-19-associated coagulopathy as the most likely cause of death. The pathophysiology of this acquired syndrome, which has recently emerged, is not fully understood, but it seems that it follows Virchow’s Triad, a fundamental model based on the interaction between the following three components: abnormalities of the blood flow; pathological changes within the blood vessels wall or endothelial surface; and circulating prothrombotic constituents, including viral RNA, factor XIa, the von Willebrand factor, thrombin–fibrin complex, cell-free DNA and platelet activation [10]. 

The limitations of this case report derive from the fact that we were not able to also determine the fibrinogen, D-dimers and activated thromboplastin time, which would have further sustained our diagnosis of COVID-19-associated coagulopathy. Moreover, it would have been useful to perform an autopsy in order to notice the macroscopic and microscopic changes within the lung parenchyma, but our legislation does not allow this in patients diagnosed with this condition. Nevertheless, this case report definitely adds valuable findings to the literature and to the current management of pediatric patients with COVID-19 since, to the best of our knowledge, it is the first pediatric case that suggested COVID-19-associated coagulopathy as the likely cause of death. Moreover, this case is of the youngest patient reported to have a COVID-19-related death in Romania, and the second case in pediatric age groups.

## 4. Conclusions

Pediatric COVID-19 is a current health burden, mostly due to the lack of knowledge in terms of symptoms, clinical course and management. In spite of its low incidence and frequent benign clinical course, COVID-19 complications such as coagulopathy might represent a leading cause of death, even in pediatric patients. Therefore, there is an urgent need for further studies in order to develop a standardized treatment protocol for COVID-19 and its associated complications, especially in children and all the reports related to this novel disease might add valuable information for researchers worldwide. 

## Data Availability

Not applicable.
